# Periodicities in the roughness and biofilm growth on glass substrate with etching time: Hydrofluoric acid etchant

**DOI:** 10.1371/journal.pone.0214192

**Published:** 2019-03-27

**Authors:** Susmita Chatterjee, Nupur Biswas, Alokmay Datta, Prasanta Kumar Maiti

**Affiliations:** 1 Institute of Post-Graduate Medical Education and Research, Kolkata, INDIA; 2 Surface Physics and Materials Science Division, Saha Institute of Nuclear Physics, 1/AF Bidhannagar, Kolkata, INDIA; University College Dublin, IRELAND

## Abstract

Adherence of the microorganism to submerged solid surfaces leads to biofilm formation. Biofilm formation modifies the surfaces in favor of bacteria facilitating the survival of the bacteria under different stressed conditions. On the other hand, the formation of biofilm has a direct adverse economic impact in various industries and more importantly in medical practices. This adherence is the reason for the failure of many indwelling medical devices. Surface biofilm adhesion is the key to biofilm growth and stability. Hence this adhesion needs to be substantially lowered to inhibit biofilm stability. Both chemical and physical properties of the surface influence biofilm formation and modulating these properties can control this formation. In this study, we have investigated the effect of Hydrofluoric acid (HF), at a specific concentration as an etchant, on the surface morphology of substrates and the growth of biofilms of *Pseudomonas aeruginosa*. and *Staphylococcus aureus*. We find that the bacterial counts on the etched surfaces undergo a periodic increase and decrease. This, on one hand, shows the close correlation between the biofilm growth and the particular roughness scale, and on the other hand, explains the existing contradictory results regarding the effects of etching on substrate roughness and biofilm growth. We propose a simple model of a sequence of hole formation, hole expansion and etching away of the hole walls to form a new, comparatively smooth surface, coupled with the preferential accumulation of bacteria at the hole edges, to explain these periodicities.

## Introduction

Biofilms consist of consortia of sessile microbial populations where heterogeneous populations of microbes remain embedded in a matrix. Within a biofilm, the bacteria are present in specific microenvironments, where they are protected from desiccation, shear forces and the actions of antimicrobials [1]. Biofilms are formed on suitable adherent surfaces. A solid surface, when immersed in water, adsorbs the neighbouring dissolved organic matter forming a ‘conditioning layer’ or ‘molecular film’. Due to the formation of this conditioning layer, physical properties such as the surface charge, wettability, hydrophobicity, and surface roughness change. These changes in their turn influence the adhesion of bacteria to the surface. Formation of the biofilm takes place in different phases [2]. In phase I the bacteria get attached to the surface reversibly over the first 1–2 hr, which is mediated through weak and mostly dispersive forces. Phase II begins approximately 2–3 hr later and is characterized by irreversible adhesion between the bacteria and the surface which is mediated through adhesive molecules secreted by the bacteria [3]. In phases III and IV, biofilm matures through the processes of chemical cross talk, production of Extracellular Poly-saccharides (EPS), and micro-colony formation. Finally, in phase V, dislodgement of planktonic bacteria from the mature biofilm takes place [4].

Surface biofilm adhesion is thus the primary condition and the key to biofilm growth and stability. Hence this adhesion needs to be substantially lowered to inhibit biofilm stability. Both the chemical and physical properties of surfaces influence biofilm formation and changing these properties can affect this formation.

The influence of the chemical composition of substrate surfaces on bacterial attachment and biofilm formation has been investigated by different research groups [5, 6]. Results show that the cell attachment to the surface can be controlled either by changing the surface chemistry of the substrate or that of the bacterial cell wall [7, 8, 9]. These different techniques of substrate surface modulation include covalent and non-covalent modification, controlled release of small molecules, and degradation of polymeric surfaces.

Modification of the surface chemistry has been found to influence the initial attachment of bacteria to the substrate, thereby reducing the surface adsorption [10, 11, 12]. For example, grafting polymer coatings on surfaces were shown to reduce attachment, leading to changes in the biofilm organization. However, chemical modulation of surfaces may not completely inhibit cell adsorption and the biofilm formation [13]. Also, chemical treatment may involve the formation of by-products that lead to adverse reactions, especially in medical circumstances.

A major technique to modify the interaction between bacteria and the surface is to change the surface roughness, and nanoporous surfaces have been utilised to this end [14]. As bacteria survive and interact in an aqueous environment, biofilm formation can be controlled by modulating the hydrophilicity of the substrates [15, 16].

In our study, we have used soda lime glass slides used for microscopy as the substrates for biofilm attachment. Although there are large variations over the actual composition of glass, silica (SiO_2_) is found to be the major component which is cross-linked to form a tetrahedral configuration [17]. Presence of this oxide makes ordinary microscopic glass slides hydrophilic, which is evident from the very low contact angle (~13° observed by us and also by others [18]) of water droplets deposited over it. This hydrophilicity of glass may be destroyed if they are kept immersed in HF solution, as the reaction of HF with the silica forms hydrophobic Hexafluorosilicate [17] and Silicon Tetrafluoride.

Besides this chemical change, HF etches the glass surface and makes it rough. Roughness introduces local ‘air-pockets’ that make the surface hydrophobic [19, 20, 21]. The roughness is expected to depend on the HF concentration and the etching time but, unfortunately, etching has produced variable results. Etching of glass surface by HF was reported to increase the surface roughness [17]. On the other hand etching of silicon surface by KOH was found to decrease the roughness, when done in combination with stirring [22]. Even a clear correlation between the etching time of a specific substrate by a specific etchant concentration and the roughness induced has not been established.

Nor is there any established correlation between the roughness produced by etching and the biofilm formation and the stability of the biofilm thus formed. While some studies reported enhancement of biofilm growth with enhanced surface roughness [22, 23], others reported just the opposite [24, 25]. This question, in all probability, is related to the type and especially the length scale of roughness induced by etching as only roughness at the length scale of micrometres will be able to affect the bacteria.

In this study, we tried to address two specific questions. (1) What is the relation between the etching time and the roughness for HF of a particular concentration on glass? (2) What is the consequent relation between the etching time/roughness and the biofilm coverage, again for the same etchant and substrate and for specific bacteria? We have shown that both these follow a periodic nature for *Staphylococcus aureus* a gram positive and for *Pseudomonas aeruginosa* a gram negative bacteria. This provides answers to both the questions and the possibility of finding a roughness for minimum coverage.

## Materials and methods

### Etching of glass surface

Microscopic glass slides (Blue Star, India) cut into 1 cm × 1 cm pieces (240 in number) were used as the surface for bacterial adhesion. The glass slides were etched chemically to modify the surface roughness. Keeping thirty such pieces as control, rest of the pieces were treated with 1:1 aqueous solution of HF of 40% strength with a time exposure of 30, 45, 60, 75, 90, 105, and 120 s. The pieces were then washed with de-ionized Millipore water (resistivity ~ 18 MΏ.cm) followed by sonication in ethanol for 5 min.

### Measurement of surface roughness and coverage

The surface roughness of each of the glass pieces, starting from the unetched (control depicted as 0s) to those exposed to HF for different time periods, was measured using a Contact Profilometer (Dektak 150 profiler, Veeco Instruments Inc). The system can be used to measure heights in 100 nm to 1 μm range [26, 27]. In this technique, a diamond stylus was moved vertically in contact with the sample and laterally across the sample with a constant 2 mg force. The position of the stylus generated an analog signal through the surface-stylus interaction (mainly dispersive forces), which was converted into a digital signal. A height map (*h*(*x*,*y*)) was generated from the scans. The glass surfaces (control and etched for different time periods, *t*) were also examined under an optical microscope (Nanonics Multiview 1000). MATLAB R2008b analysis of optical images was done to find out etched area fraction *A*(*t*). *A(t)* corresponds to the ratio of etched area to the total observed area of glass substrates and hence it is unit less quantity. *A(t)* was calculated from 5 areas of each sample where each area corresponds to 182 μm × 99 μm.

### Growth of bacteria on glass surface

Clinical isolates of *S*. *aureus* and *P*. *aeruginosa* collected from biofilms formed on renal catheters, were grown on substrates etched for the above mentioned times and were studied to see the effect of etching time on growth. Bacteria were grown overnight in tryptic soy broth (Himedia, India) at 37°C and then diluted in same media to an optical density of 0.5 at 600 nm. The diluted culture was poured over the etched glass plates and incubated at 37°C for 48 hrs. The glass plates were aseptically removed and washed with phosphate buffered saline (PBS, pH ~ 7.2) by shaking at 180 rpm for 1 min. This well-established step eliminates all free floating bacteria and only sessile forms remain attached on the glass surface [28]. Different sets were prepared for microscopy and colony count.

### Colony forming unit (CFU) counting

The substrates with their surfaces bearing biofilms were immersed in PBS and sonicated to remove all attached cells as the standard protocol to remove biofilm from a surface in order to always start from an identically uncontaminated condition. The total number of viable colonies (*N*) grown on the entire glass surfaces (1 cm × 1 cm) was obtained by the method of Miles and Mishra [29]. We have quantified the dependence of growth on etching time by determining *N*(*t*). Data averaged over five different isolates, each of P. aeruginosa and S. aureus for each etching time point is presented here.

### Scanning electron microscopy

Biofilms grown on etched surfaces were observed under a scanning electron microscope (SEM, FEI quanta 200F). The glass pieces with attached bacterial cells were covered with 2.5% glutaraldehyde and kept for 3 hrs. at 4°C. They were then washed thrice with 0.1M phosphate buffer, passed once through a graded series of ethanol consisting of 25%, 50%, 75% and twice through 100% ethanol each for 10 min. The slides were then transferred to critical point drier and kept overnight.

The images were analysed by ImageJ software version 1.47t (http://imagej.nih.gov/ij, freeware by National Institute of Health, US).

## Results and discussion

### A. Periodic evolution of roughness with etching time

#### Optical microscopy

**Fig 1:**
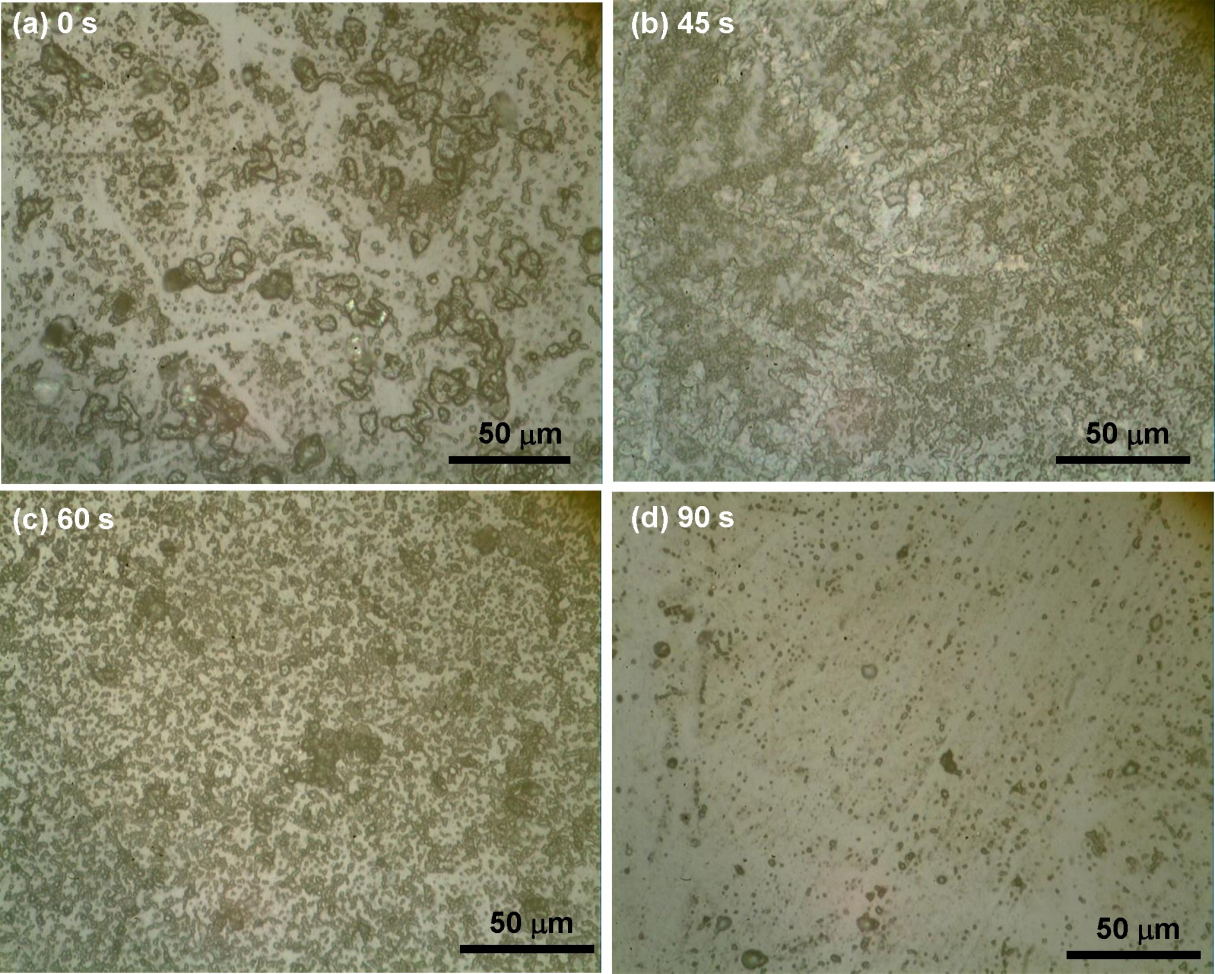
Effect of Hydrofluoric (HF) acid etching on glass substrates. Optical microscopic image of glass substrates etched by HF for (a) 0 s, (b) 45 s, (c) 60 s and (d) 90 s. The sample having 0 s etching duration is the control sample.

**Fig 2:**
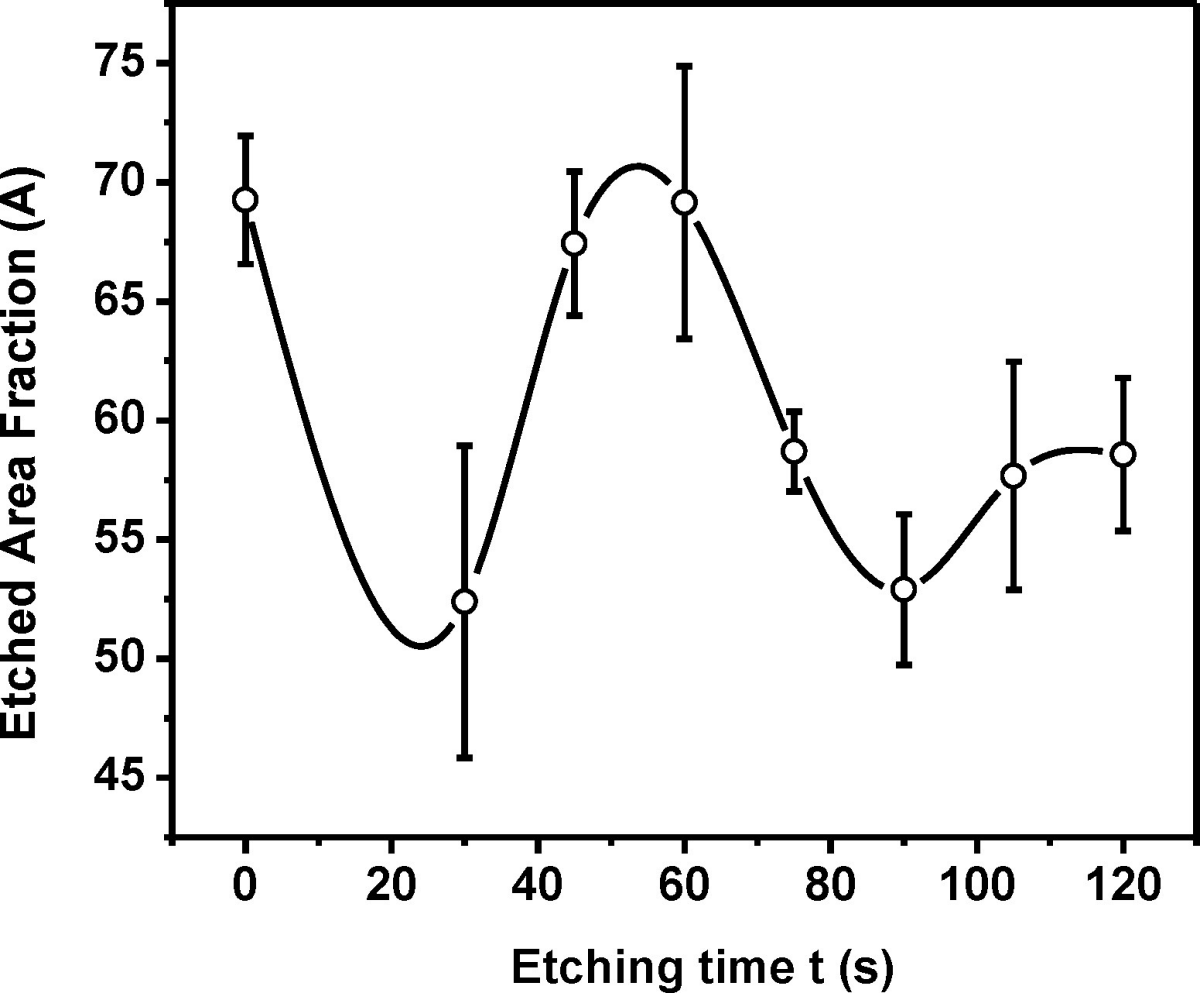
Variation in etched area fraction with etching time. Etched area fractions (*A*) corresponding to the ratio of etched area to the total observed area of glass substrates shown in Fig 1 with additional samples (etching durations of 30 s, 75 s, 105 s, and 120 s), is plotted against etching time (*t*). Area fractions were calculated from at least 5 areas (each of dimension 182 μm × 99 μm) of a given sample. Average values and error bars are shown in the graph. Average value points are connected by a spline curve for visual aid only.

When observed under an optical microscope, i.e., at the local, micrometre scale, the etched surfaces did not appear to be rough but consisted of holes of different sizes on the surface (Fig 1). It is important to note that *A*(*t*) shows (Fig 2) a periodic behaviour with an increase in etching time, with a minimum at 30 s, followed by a maximum at 60 s, and the next minimum at 90 s. Control data presented as 0s. Data points had been connected by spline as a visual guide.

#### Profilometry

**Fig 3:**
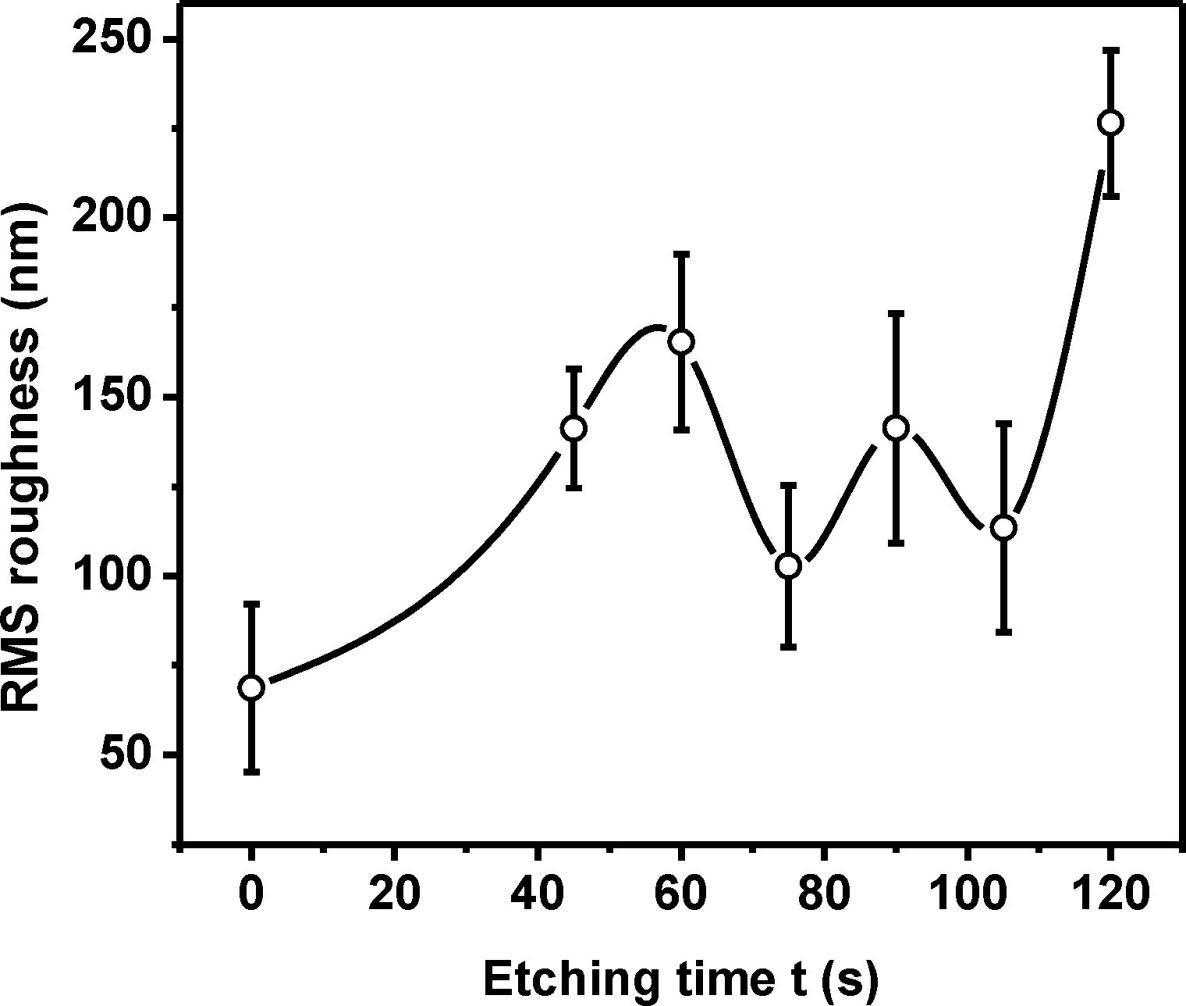
Variation of surface roughness with etching time. Roughness was measured by profilometry, scanning at least 5 areas of the samples shown in Fig 1 with additional samples (etching durations of 30 s, 75 s, 105 s, and 120 s), averaging over 40 profiles in each case. Data points are connected by spline curve for visual guide only.

At this point, we realized that the ‘roughness’ of the etched surface is of the order of 100 nm. This led us to use the Profilometer, which can measure such large fluctuations in height. Scans were carried over a 150 μm × 150 μm area at a height resolution of 44 nm and averaged over 40 profiles. We extracted the data and analyzed it by Vision and Origin pro 8.5 and Matlab R2008b software. From an analysis of *h*(*x*,*y*) we got both the mean height (<*h*>) of the bacterial biofilm and the root mean square height fluctuation or roughness, ρ. We then determined ρ(*t*) from data collected for the different etching times *t*. We present ρ(*t*) in Fig 3 as an answer to our first question.

As evident from this figure, with an increase in *t* the roughness of the surface increases uniformly but non-linearly until 60 s of etching. This general trend of non-linear increase is in consistence with previous reports [8] though the exact relation depends on glass composition. However, after 60 s there is a sudden decrease in roughness at 75 s. This trend persists till 90 s of etching time and then the roughness again starts to increase, as evidenced from the 105 s and 120 s data. The data points have been connected by spline to bring out the periodic nature. This periodicity, which matches quite well with that in *A*(*t*), has never been reported before, most probably because such a systematic study over such long periods of etching have not been carried. It perhaps explains the existing conflicting reports on the correlation of etching time with roughness.

### B. Periodicity in bacteria growth

**Fig 4:**
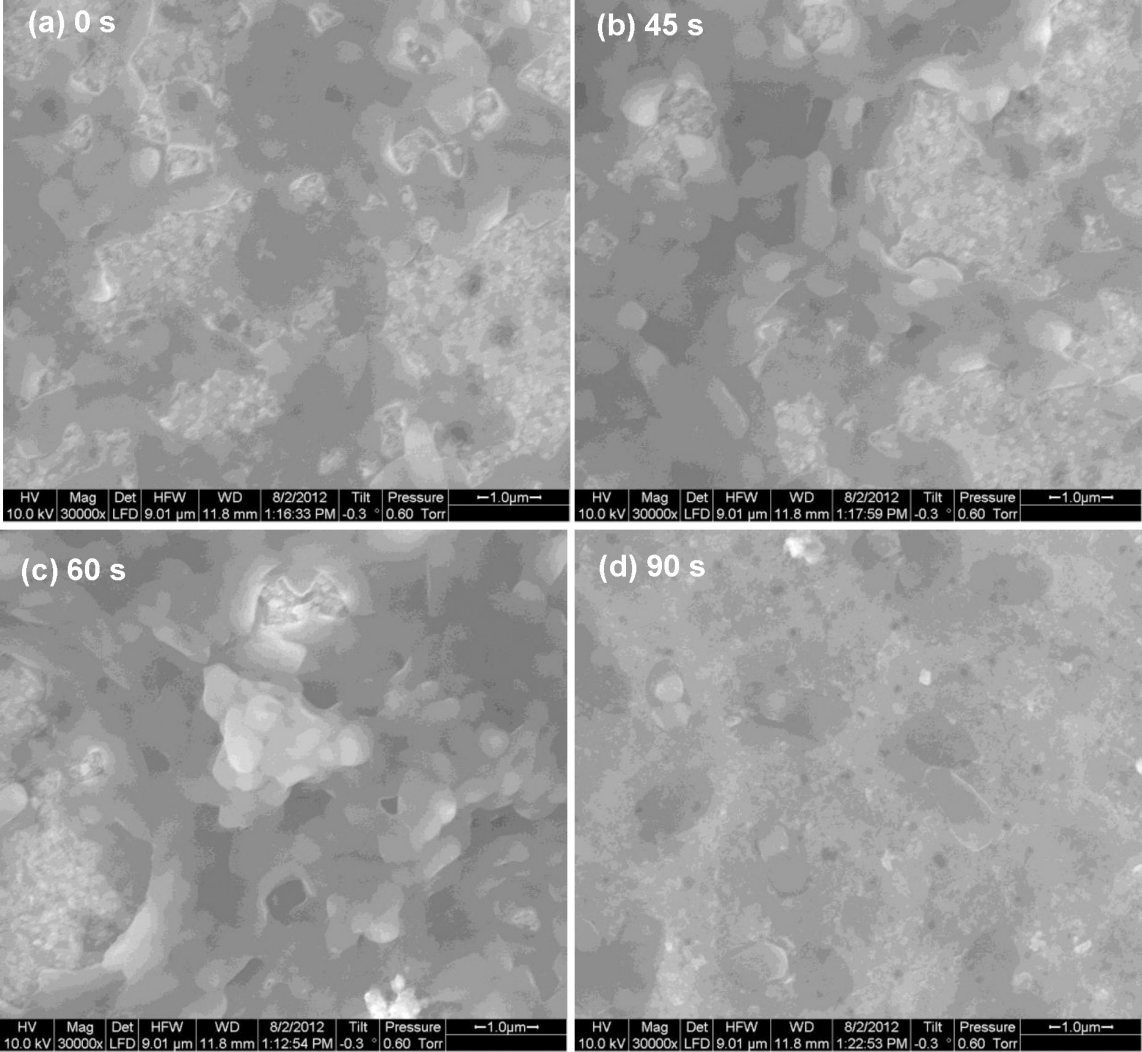
Effect of substrate surface etching on biofilm growth. Scanning Electron Microscopic (SEM) image of *Pseudomonas aeruginosa* biofilm on glass surfaces etched for (a) 0 s (b) 45 s (c) 60 s and (d) 90 s. The sample at 0 s is the control sample.

**Fig 5:**
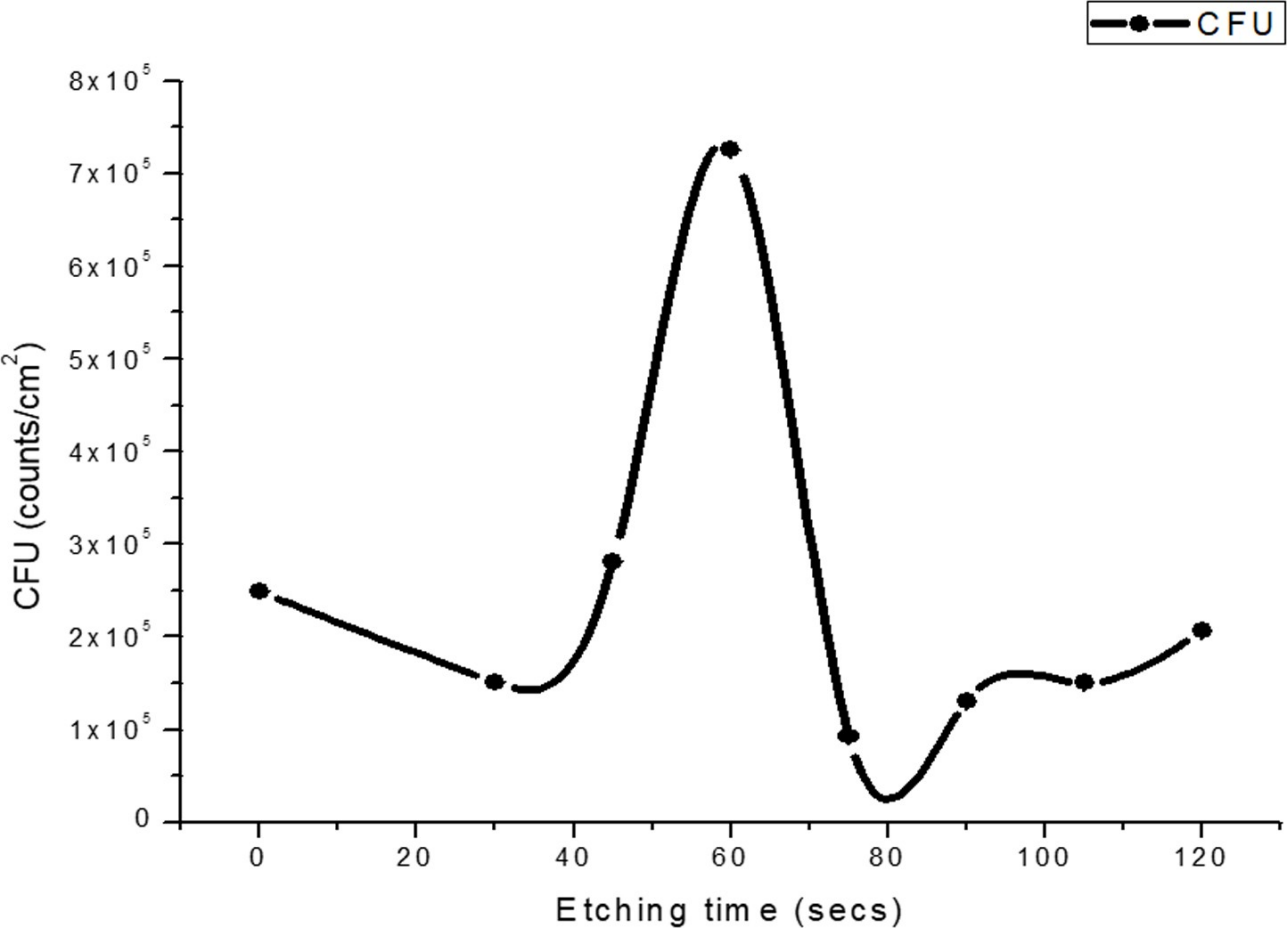
*Pseudomonas* biofilm growth with etching time. Colony Forming Unit (CFU) of *P*. *aeruginosa* (five isolates) from the samples shown in Fig 4 with additional samples (etching durations of 30 s, 75 s, 105 s, and 120 s). The data at 0 s is the control data. Averages taken over 5 isolates of the bacteria for each etching time point are shown. The spline joining the averages is for visual aid only.

With an increase in etching time, disruption of biofilm architecture and continuity of *P*. *aeruginosa* were noticed in images obtained by scanning electron microscopy (SEM) (Fig 4). Hence, a distinct correlation between the increase in surface roughness and adherence of bacteria were observed in our study. The nature of *N*(*t*) is shown in Fig 5. The errors or fluctuations in the CFU in both this and the succeeding instance for *S*. *aureus* are about five orders of magnitude lower than the average values and hence, naturally, cannot be observed in the plots. The CFU count (Fig 5) shows an increase with the etching time but this growth is considerably non-linear with a peak at *t* = 60 s. After this peak, the count starts going down, reaches a minimum at 90 s, and then rises again, more or less consistent with the roughness behaviour as shown in Fig 3.

**Fig 6:**
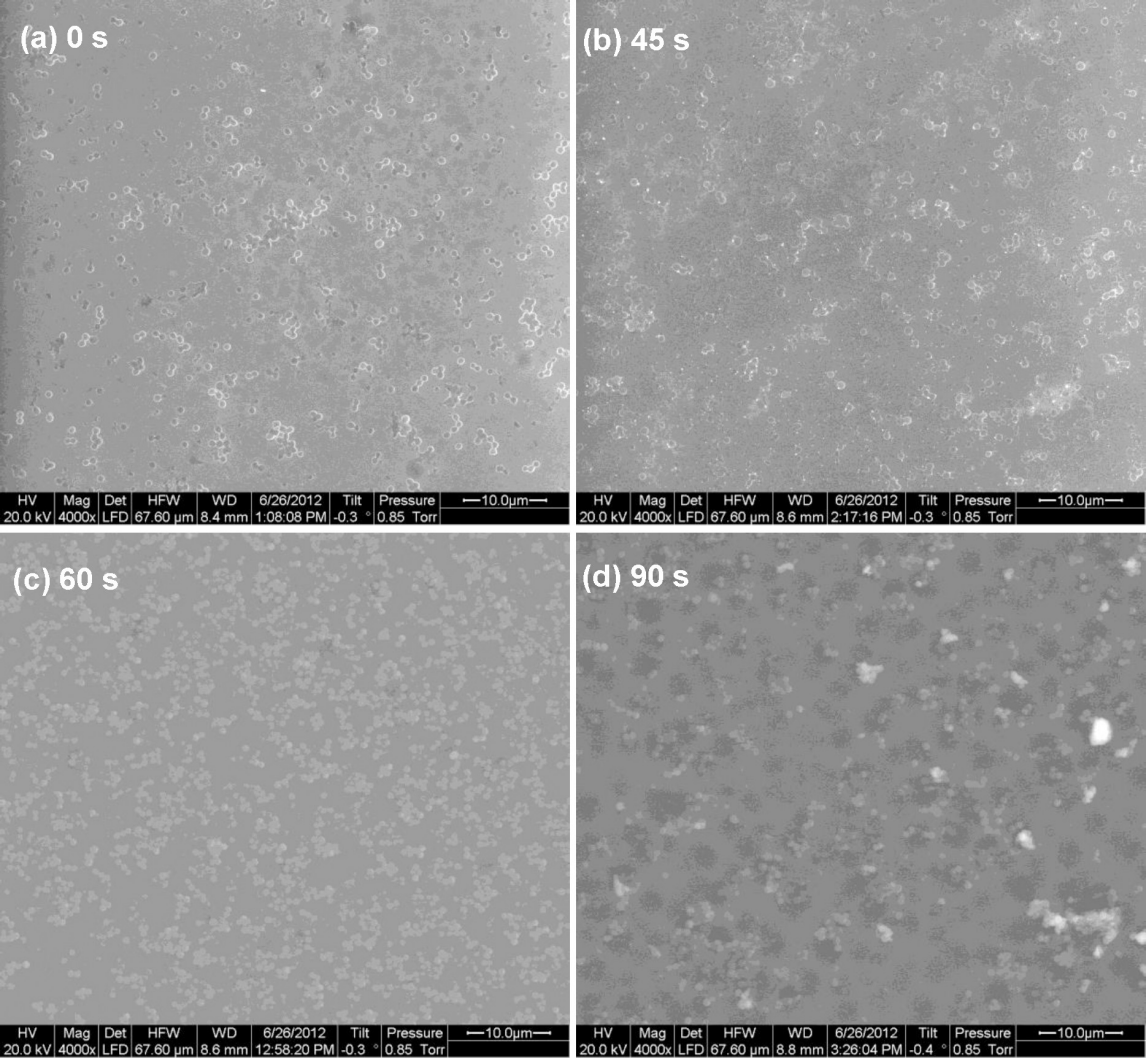
Effect of substrate surface etching on biofilm growth. SEM image of Staphylococcus aureus biofilm grown on glass surfaces etched for (a) 0 s (b) 45 s (c) 60 s and (d) 90 s. The sample at 0 s is the control sample.

**Fig 7:**
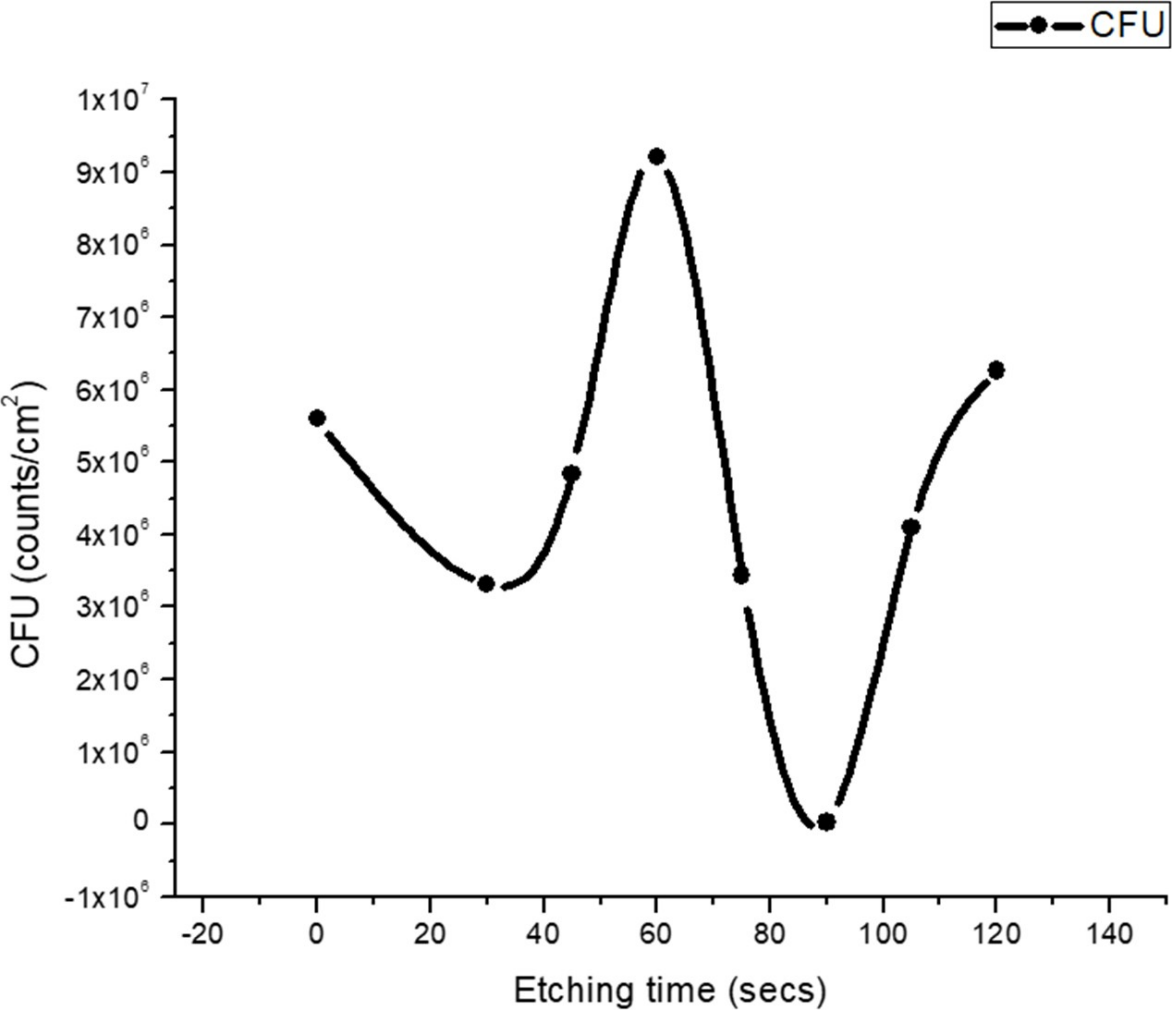
*Staphylococcus* biofilm growth with etching time. Colony Forming Unit (CFU) of *S*. *aureus* (five isolates) from the samples shown in Fig 6 with additional samples (etching durations of 30 s, 75 s, 105 s, and 120 s). The data at 0 s is the control data. Averages taken over 5 isolates of the bacteria for each etching time point are shown. The spline joining the averages is for visual aid only.

As against three-dimensional multi-layered biofilm of *P*. *aeruginosa*, biofilms formed by *S*. *aureus* on glass surface were flattened and monolayered (Fig 6) throughout the range of HF etching time. However, in spite of this morphological distinction, the behaviour of *N*(*t*) is very similar, as shown in Fig 7. This suggests that, at least for this pair of etchant and substrate, the periodic nature of the dependence of growth on etching time is a general feature and follows the periodicity of the roughness with etching time.

### C. A proposed model for growth dependence

**Fig 8:**
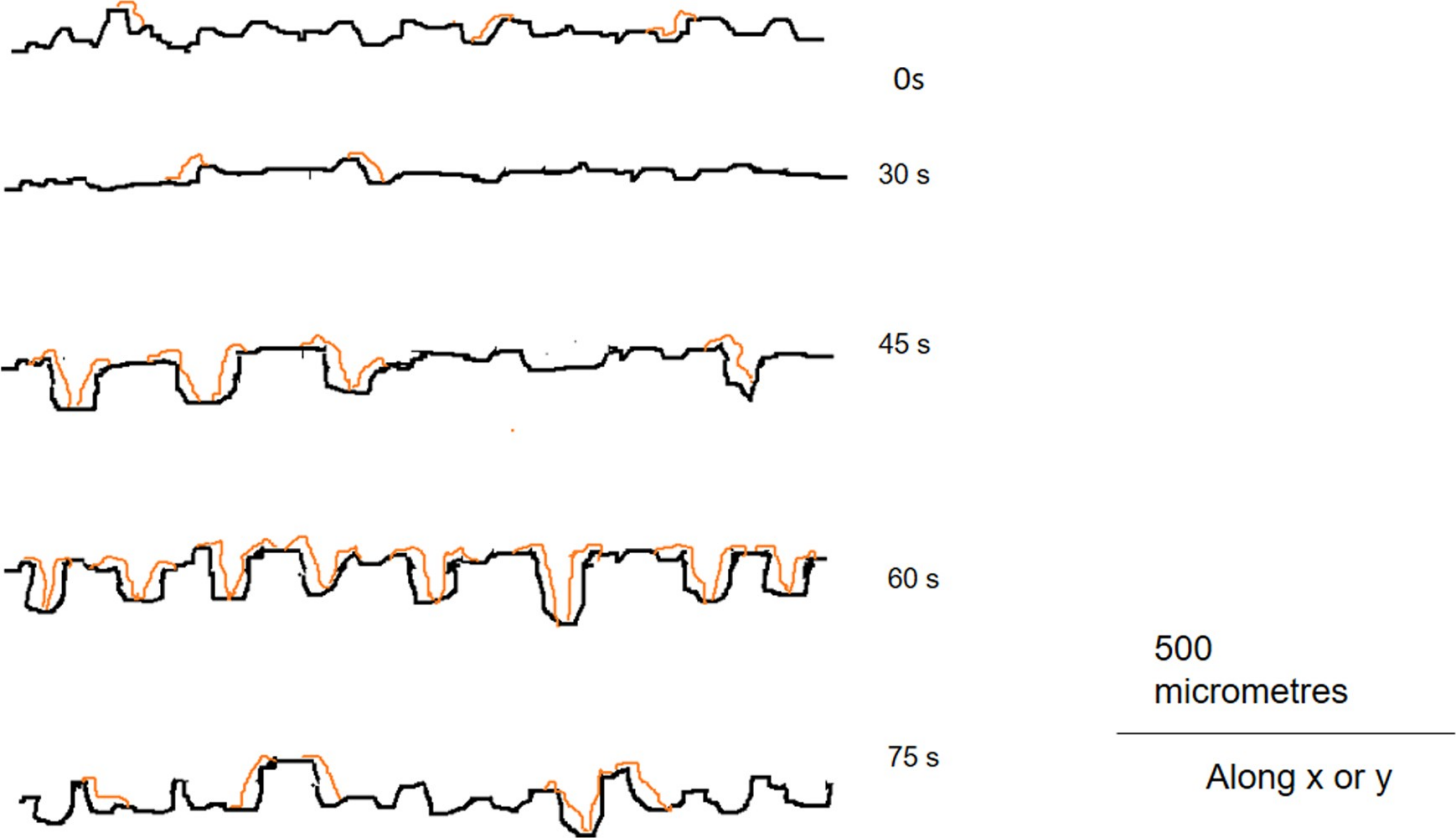
Cartoon showing a schematic representation of the growth of biofilms on glass surfaces etched for different times from a cross-sectional view. The black line represents the glass surface, the red line shows bacterial biofilm. The nominal length scale is indicated.

We propose that the substrate surface is periodically roughened and smoothened by the action of hydrofluoric acid. The action of the etchant is giving rise to two kinds of 'roughening' due to the local in-homogeneities at the glass surface. The first kind is at a nanometer or even sub-nanometer scale of height, i.e., it is a local fluctuation in height. The other type of roughness is much larger and creates holes at the scale of 10 or even 100 nm. The first type of roughness does not differ much on the top or within the hole and has no effect on *A* or *N*. It is the second type of roughness which affects these quantities. A possible model for the evolution of this roughness which we measured by profilometry and designated as *ρ* is shown schematically in the cartoon of Fig 8. As the etching time is increased the holes increase in number and size. However, after some time the walls of these holes are also etched away, and the surface regains its smoothness to an extent. With further etching, the holes reappear, and the process is repeated. This explains the periodic nature of *ρ*(*t*). These holes again provide the test bacteria shelters against the action of shearing forces. Also, in the edges of the holes the surface free energy is high which further facilitates bacterial colonization. Thus we observe a close correlation between *ρ*(*t*) and *N*(*t*) and the periodic behaviour of biofilm growth with etching time is explained.

## Conclusion

In this communication, we have investigated the effect of HF, of a specific concentration as etchant, on the surface morphology of a substrate and on the growth of biofilms of *P*. *aeruginosa*. and *S*. *aureus* We have shown, through consistent results from diverse techniques such as profilometry and optical microscopy, and colony forming unit counting and scanning electron microscopy, that respectively, (a) the 100 nm– 250 nm scale of roughness of and (b) the bacterial count on, the etched surface undergo a periodic increase and decrease. This on one hand, shows the close correlation between the biofilm growth and the particular roughness scale, and on the other hand explains the existing contradictory results regarding the effects of etching on substrate roughness and on biofilm growth. We have put forward a simple model of a sequence of hole formation, hole expansion and etching away of the hole walls to form a new, comparatively smooth surface, coupled with the preferential accumulation of bacteria at the hole edges, to explain these periodicities.
